# Successful orthotopic heart transplantation following total aortic replacement in a patient with Marfan syndrome: case report

**DOI:** 10.1186/s44215-026-00249-2

**Published:** 2026-04-17

**Authors:** Tatsuya Tago, Masatoshi Akiyama, Kentaro Yuda, Kota Itagaki, Katsuhiro Hosoyama, Koki Ito, Yusuke Suzuki, Shintaro Katahira, Goro Takahashi, Kiichiro Kumagai, Yoshikatsu Saiki

**Affiliations:** 1 Division of Cardiovascular Surgery, Tohoku University Hospital, 1-1, Seiryo-machi, Aoba-ku, Sendai, Miyagi, 980-8574 Japan; 2 Division of Cardiovascular Surgery, NHO Sendai Medical Center, Sendai, Japan

**Keywords:** Heart transplantation, Marfan syndrome, Total aortic replacement, Ischemic cardiomyopathy, Left ventricular assist device, Strategy, Decision-making, Risk mitigation

## Abstract

**Background:**

Aortic dissection and aneurysm rupture are leading causes of death in patients with Marfan syndrome. Due to the risk of such vascular complications, the indication for heart transplantation in this population remains controversial. Previous reports, however, have demonstrated that heart transplantation in patients with Marfan syndrome can result in good long-term survival comparable to that in patients without Marfan syndrome. Here, we present a case of successful orthotopic heart transplantation after total aortic replacement in a patient with Marfan syndrome.

**Case presentation:**

A 41-year-old man with Marfan syndrome, who had been managed conservatively for type B aortic dissection, developed acute type A dissection involving both coronary arteries at age 30, resulting in myocardial infarction. He underwent aortic root replacement with a mechanical valve. Thereafter, he was repeatedly hospitalized for heart failure due to ischemic cardiomyopathy. Three years later, at age 33, he was referred to our institution for heart transplant registration because of progressively deteriorating cardiac function and frequent episodes of intractable lethal arrhythmias. He underwent replacement of the ascending aorta and aortic arch, along with bioprosthetic aortic valve replacement. His immediate postoperative course was complicated by hemodynamic decompensation, necessitating support with an extracorporeal left ventricular assist device. One year later, he underwent a thoracoabdominal aortic replacement and bridge-to-bridge implantation of a left ventricular assist device. Three years later, at age 37, he underwent replacement of the durable device because of a pump pocket infection. After 7 years on the transplantation list, he underwent successful orthotopic heart transplantation from a suitable donor at the age of 41.

**Conclusions:**

This rare case report describes a patient with Marfan syndrome who underwent orthotopic heart transplantation after total aortic replacement, followed by multiple surgical procedures, including extracorporeal and durable left ventricular assist device implantations, over an extended period. Although total aortic replacement was challenging in the context of complex coexisting cardiovascular disease, this approach enabled subsequent heart transplantation after mitigating the potential risk of Marfan syndrome-associated cardiovascular events.

**Supplementary Information:**

The online version contains supplementary material available at 10.1186/s44215-026-00249-2.

## Background

Cardiovascular disease accounts for more than 90% of premature deaths in patients with Marfan syndrome [1]. Due to the risk of vascular complications such as aortic dissection or aneurysm rupture, the indication for heart transplantation in these patients remains controversial. Although prophylactic ascending aortic replacement during orthotopic heart transplantation has been reported in patients with Marfan syndrome and borderline aortic dilatation [2], heart transplantation after total aortic replacement is rarely described. Here, we report a case of successful orthotopic heart transplantation in a patient with Marfan syndrome following total aortic replacement and subsequent staged support with extracorporeal and durable left ventricular assist device (LVAD) implantations.

## Case presentation

**Fig. 1:**
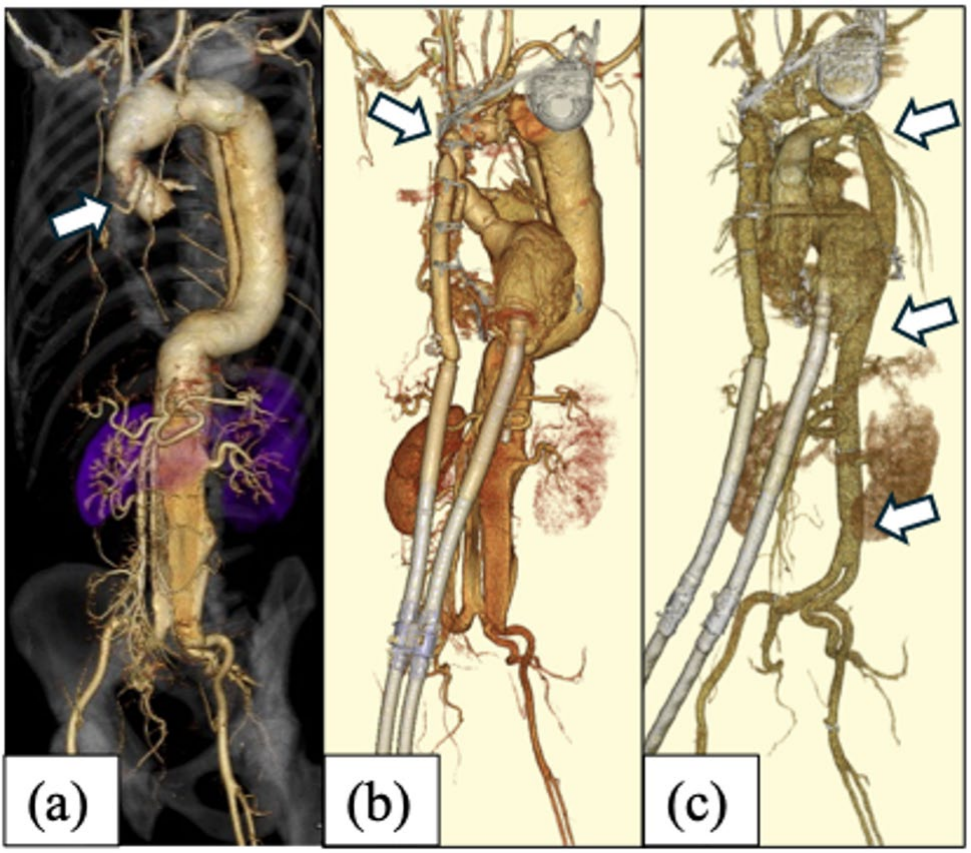
Three-dimensional computed tomography (3D-CT) images demonstrating the staged progression to total aortic replacement. (**a**) 3D-CT image obtained at the time of transfer to our hospital. The aortic root had been replaced with a composite graft incorporating a mechanical prosthetic valve, and both coronary arteries were reconstructed using the Piehler technique. Marked enlargement of the dissecting aorta from the aortic arch to the terminal aorta was observed. (**b**) 3D-CT image after redo aortic valve replacement with a bioprosthetic valve, total aortic arch replacement, and subsequent implantation of an extracorporeal left ventricular assist device (LVAD). (**c**) 3D-CT image obtained after thoracoabdominal aortic replacement extending to the bilateral common iliac arteries under extracorporeal LVAD support. At this stage, total aortic replacement had been completed

A 41-year-old man with Marfan syndrome was initially managed conservatively for type B aortic dissection (DeBakeyⅢb) at age 28. At age 30, he developed an acute type A dissection (DeBakey II) involving both coronary arteries, which led to myocardial infarction, and underwent aortic root replacement with a mechanical valve. Thereafter, he was repeatedly hospitalized for heart failure due to ischemic cardiomyopathy. Although a cardiac resynchronization therapy-defibrillator was implanted at age 32, his heart function gradually declined, and he began to experience frequent episodes of intractable lethal arrhythmias. He was subsequently transferred to our hospital at age 33 for heart transplant registration. At transfer, the patient had a dissecting aortic aneurysm with a maximum diameter of 48 mm in the distal aortic arch and 60 mm in the thoracoabdominal aorta (Fig. 1a). No other contraindications to heart transplantation were identified. Given the risk of rupture and the long-term post-transplant prognosis, we determined that both aortic arch replacement and thoracoabdominal replacement were necessary before applying for heart transplantation. Because a single-stage procedure would have been excessively invasive, we selected a staged approach. In anticipation of potential LVAD implantation as a bridge to transplantation, the previously implanted mechanical valve should be replaced with a bioprosthetic valve; therefore, the first-stage procedure consisted of redo aortic valve replacement (AVR) and total aortic arch replacement, followed by thoracoabdominal aortic replacement as the second stage.

In the year of the transfer, the patient underwent redo AVR using a 19-mm CEP magna EASE (Edwards Life Science, Irvine, CA, USA) and total aortic arch replacement using a 22-mm J Graft Shield Neo (Japan Lifeline Co, Ltd, Tokyo), along with placement of a 6-cm long 20-mm Hemashield Platinum Woven Double Velour graft (Intervascular SAS, La Ciotat, France) as a conventional elephant trunk. Despite low cardiac function, weaning from cardiopulmonary bypass was uneventful. Postoperatively, however, left heart failure and pulmonary edema developed. Although transplant approval had not yet been granted, the patient was classified as INTERMACS profile 1, and an emergency extracorporeal LVAD (HAS-CFP, MERA Senko, Tokyo) incorporating an artificial lung (Capiox FX25, Terumo, Tokyo) was urgently implanted (Fig. 1b), which rapidly improved the pulmonary edema.

Approximately 3 months later, gastrointestinal bleeding occurred but resolved with endoscopic intervention and anticoagulant adjustment, allowing us to proceed to the planned second stage. The following year, thoracoabdominal aortic replacement extending to the bilateral common iliac arteries was performed under extracorporeal LVAD support (Fig. 1c). Given the bleeding risk associated with LVAD support, cerebrospinal fluid drainage and epidural catheters were not placed. The Adamkiewicz artery was identified preoperatively and reconstructed intraoperatively, and no postoperative paraplegia occurred.

Intraoperative bleeding control under full heparinization was a major consideration. A total of 22,000 units of heparin and 100 mg of protamine were administered. Total blood loss was 5696 mL, requiring 2520 mL of red blood cells, 1920 mL of fresh frozen plasma, 600 mL of platelet concentrates, and 180 mL of allogeneic cryoprecipitate. Activated clotting time control was maintained between 500 and 1500 s. Bleeding from dissection surfaces after protamine reversal was less than anticipated. Hemostasis was achieved through meticulous surgical techniques and appropriate transfusion support.

Following thoracoabdominal aortic replacement, the patient’s heart transplant application was approved. To promote rehabilitation, conversion from an extracorporeal LVAD to an implantable LVAD was planned to prevent disuse. However, an infection developed at the drain insertion site and was treated with negative-pressure wound therapy (NPWT). During the follow-up, multidrug-resistant *Pseudomonas aeruginosa* was detected in central venous catheter cultures, necessitating long-term antibiotic therapy. After confirming negative cultures, bridge-to-bridge LVAD conversion using a DuraHeart device (Terumo Heart, Inc, Ann Arbor, MI, USA) was performed. At that time, there was no evidence of infection involving the extracorporeal LVAD pump. However, the omentum was prophylactically trimmed and positioned to fully cover the pump, inflow and outflow grafts, and driveline. Six months later, repeated pump pocket hematoma and multiple hematoma evacuations caused delayed wound healing. Despite the sternum being closed, part of the outflow graft was exposed from the lower portion of the midline of the chest. The omentum was again trimmed to cover the exposed graft and NPWT was initiated. To prevent recurrent exposure, a muscle flap was harvested to fill the dead space around the graft, followed by split-thickness skin grafting from the thigh. Despite requiring multiple procedures, wound healing progressed favorably with no further device exposure.

**Fig. 2:**
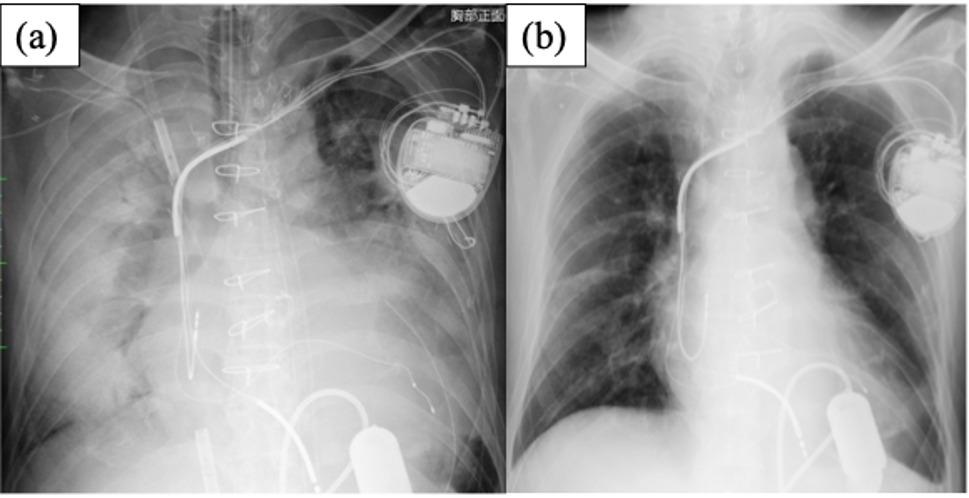
Chest radiographs after Jarvik 2000 implantation. (**a**) Chest X-ray obtained immediately after initiation of veno-venous extracorporeal membrane oxygenation (VV-ECMO) following Jarvik 2000 implantation for pneumonia-associated acute respiratory distress syndrome. (**b**) Chest X-ray obtained after V-V ECMO withdrawal. These images demonstrate that severe pulmonary complications were successfully managed with a lung-protective strategy using ECMO, allowing the patient to undergo heart transplantation

Approximately 3 years after the DuraHeart implantation, the device was replaced with a Jarvik2000 (Jarvik Heart, Inc, New York, NY, USA) due to pump-pocket infection. The postoperative course was complicated by acute respiratory distress syndrome secondary to bacterial pneumonia with pulmonary hemorrhage, requiring veno-venous extracorporeal membrane oxygenation support for 1 month (Fig. 2). The patient recovered from these complications, and after 7 years on the transplantation list, a suitable donor became available.

**Fig. 3:**
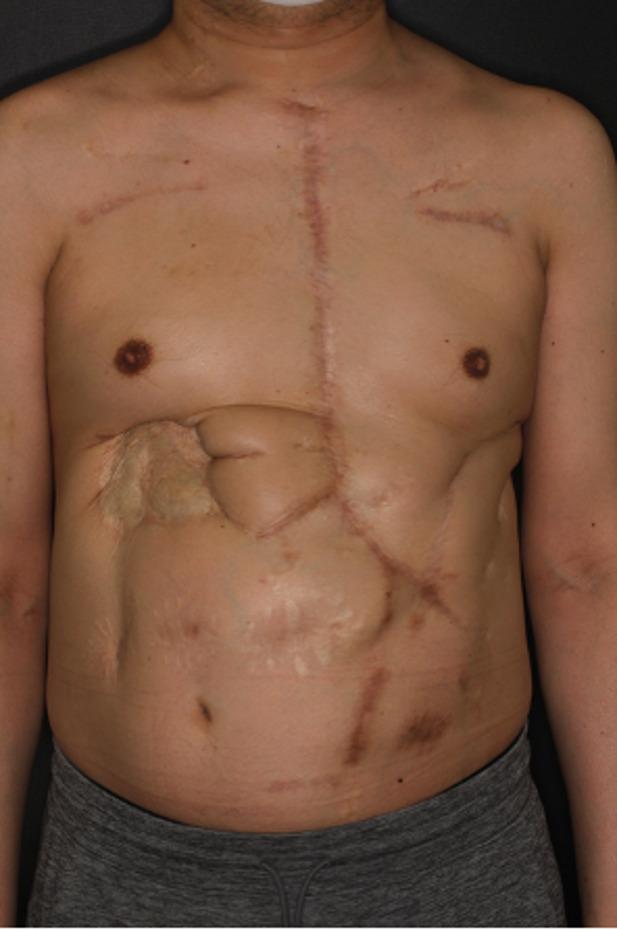
Surface photograph of the patient’s chest after heart transplantation. This image demonstrates that, following multiple reconstructive surgeries due to wound infection, care was taken during the median sternotomy for heart transplantation to avoid extending the incision into the muscle flap

**Fig. 4:**
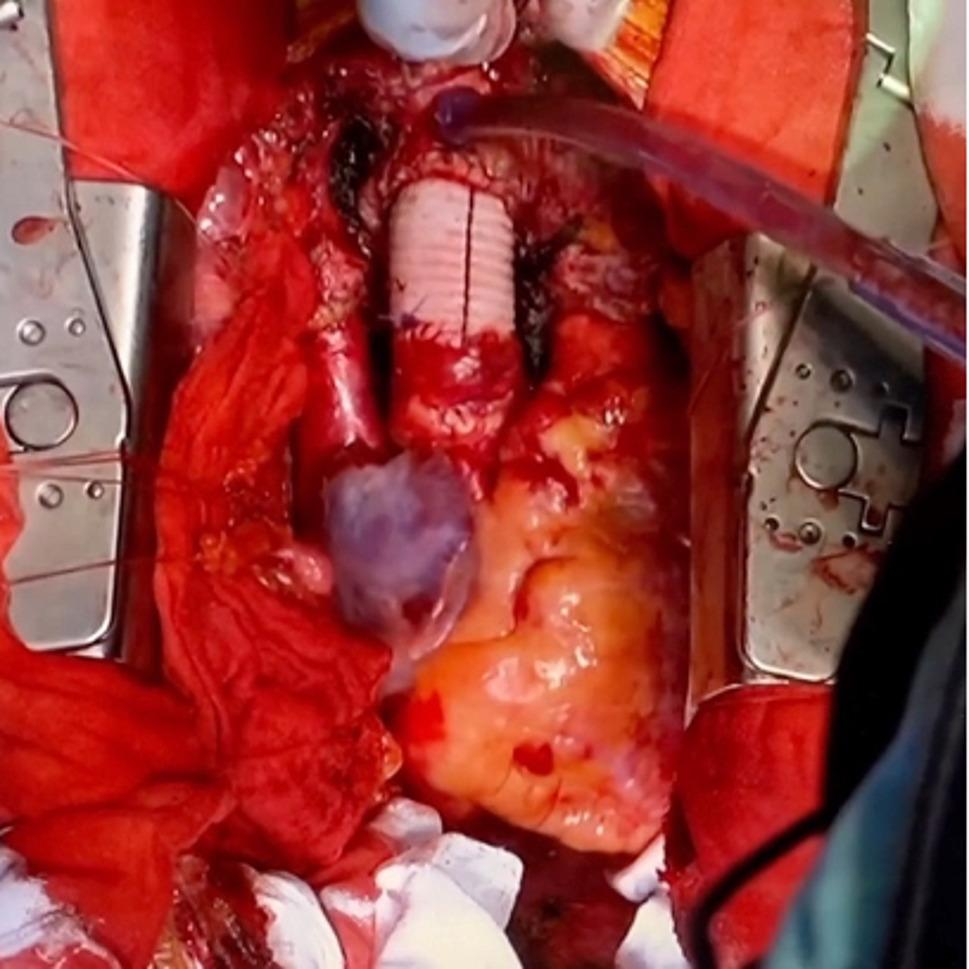
Intraoperative photograph after completion of orthotopic heart transplantation. Despite the prolonged donor heart ischemic time due to unplanned hypothermic circulatory arrest to replace an injured ascending aortic prosthetic graft, the patient was successfully weaned off cardiopulmonary bypass with low-dose catecholamine support and excellent contractility

Pre-transplant computed tomography showed no abnormalities at previous aortic surgical sites. Sternotomy was carefully performed to avoid injury to the skin flap (Fig. 3). Because of severe adhesions, significant bleeding from the ascending aortic graft occurred during the dissection, necessitating hypothermic circulatory arrest (25℃) and redo ascending aortic replacement with a 22 -mm Gelweave (Terumo Aortic, Glasgow, Scotland) under open distal anastomosis with selective cerebral perfusion. Orthotopic heart transplantation was then completed using the bicaval technique (Fig. 4). Despite the long donor-heart ischemic time (449 min), the patient was weaned from cardiopulmonary bypass with low-dose catecholamine support, and the postoperative course was uneventful. He was discharged home 60 days after transplantation.

No clinically significant rejection has been observed during the follow-up (International Society for Heart and Lung Transplantation heart biopsy grading scale: Grade 1 A).Although renal dysfunction related to long-term immunosuppression and BK virus nephropathy required initiation of dialysis, his condition remains stable 3 years after transplantation (Supplementary data 1&2).

### Discussion and conclusions

Staged surgical strategies in patients with Marfan syndrome, such as simultaneous heart transplantation with ascending aortic replacement or treatment of aortic disease after heart transplantation resulting in total aortic replacement, are occasionally reported. The present case is unique in that heart transplantation was performed after completion of total aortic replacement, thereby addressing the risk of rupture of the residual chronic dissecting aortic aneurysm.

Aortic rupture or dissection is a well-recognized complication after orthotopic heart transplantation in patients with Marfan syndrome. Botta et al. reported a higher incidence of such complications in Marfan patients than in those without connective tissue disorders [3]. Post-transplant increases in blood pressure and cardiac output, together with chronic immunosuppression, may exacerbate aortic fragility, contributing to ongoing controversy regarding heart transplantation in this population (Table 1) [4].

**Table 1:**
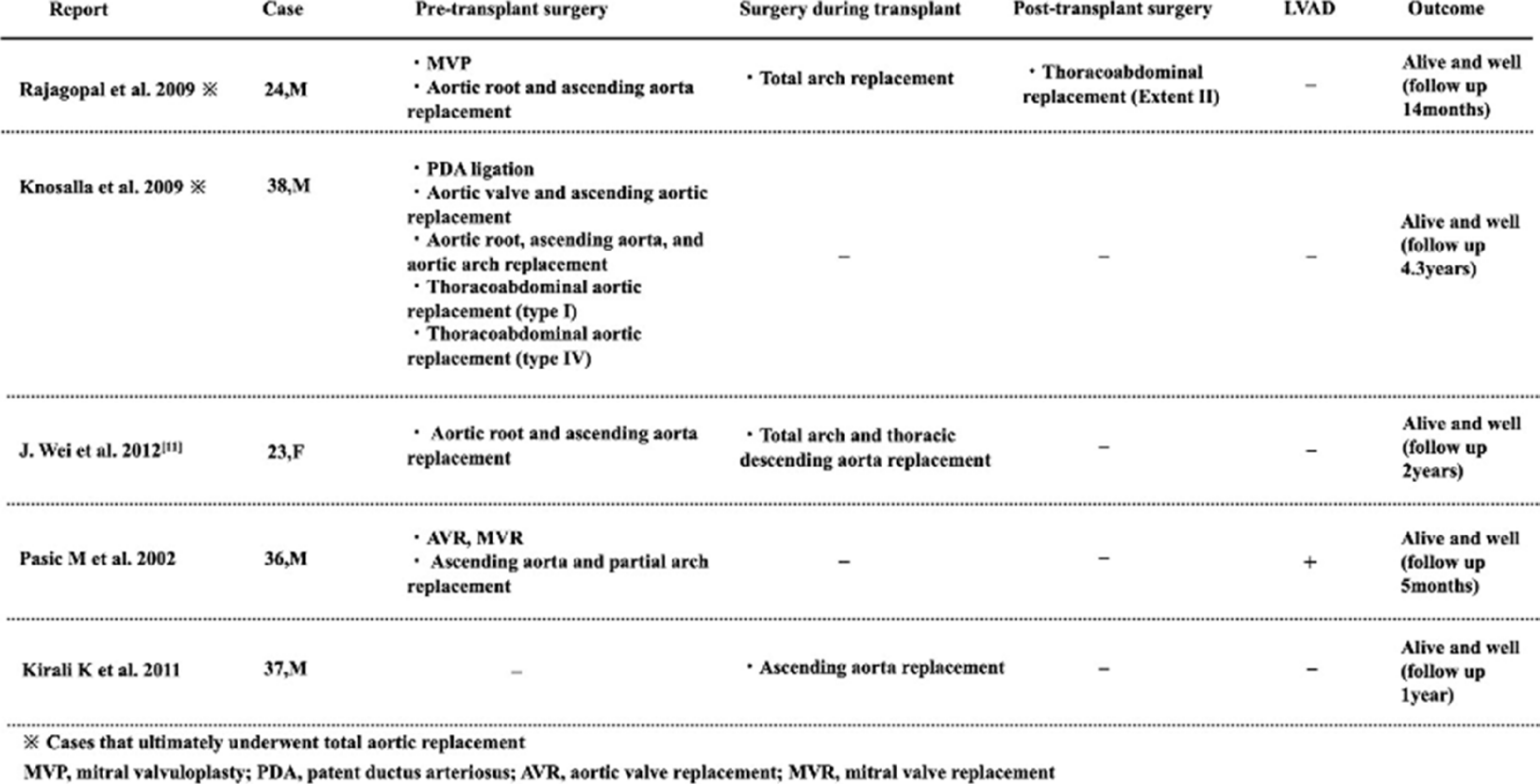
Previous reports of total or partial aortic replacement and heart transplantation in patients with Marfan syndrome

Kesler et al. suggested that reluctance to list Marfan patients for heart transplantation may be justified, as vascular complications can adversely affect post-transplant outcomes [5]. In their series of 11 Marfan patients, operative mortality was 9.1%, the incidence of post-transplant thoracic aortic dissection was 40%. On the other hand, other reports have demonstrated favorable long-term survival outcomes [6, 7]. Knosalla et al. described 10 Marfan patients with no perioperative mortality, reporting survival rates of 80% at 1 year, 64% at 10 years. Two deaths, however, were attributed to aortic dissection and aortic rupture at 3.8 and 12.1 years post-transplant, respectively [6].

Thus, even when overall survival after heart transplantation in patients with Marfan syndrome is comparable to that of patients without Marfan syndrome, late cardiovascular complications remain a major concern. Kirali et al. suggested that prophylactic replacement of a borderline–dilated ascending aorta (approximately 45 mm) at the time of transplantation may improve long-term survival by preventing later vascular complications [2]. Rajagopal et al. described a Marfan patient with end-stage heart disease and chronic aortic dissection who underwent combined orthotopic heart transplantation and total aortic arch replacement, followed 4 months later by second-stage thoracoabdominal aortic replacement [8]. Pasic et al. reported successful bridging to heart transplantation 217 days after combined aortic surgery and LVAD implantation [9]. Although evidence-based guidelines for preventive aortic strategies in this population are lacking [10], our strategy may be reasonable in selected Marfan patients with progressive residual aortic disease.

Delayed risks following total aortic replacement also warrant careful attention. Long-term immunosuppressive therapy and extensive prosthetic grafting require careful monitoring for graft infections and may justify continued prophylactic oral antibiotics. Because connective tissue disorders are systemic, new dissections or aneurysms may develop in unreplaced peripheral arteries and branch vessels, requiring rigorous imaging follow-up. In our case, computed tomography at 3 years post-transplant showed no coronary artery disease, graft infection, aortic dissection, or peripheral aneurysm formation.

Aortic risk in Marfan syndrome varies by the type and location of mutations in the *FBN1* gene [11]. Haploinsufficiency mutations, which reduce production of normal fibrillin-1, are associated with a higher risk of aortic dissection and cardiovascular mortality than dominant-negative mutations, in which abnormal and normal fibrillin-1 coexist. Therefore, stricter blood pressure management from an earlier stage is recommended in this subgroup. Mutations in exons 24–32 of *FBN1*, often associated with the neonatal subtype of Marfan syndrome, may also cause early ascending aortic enlargement in adults. Patients with these genetic subtypes may particularly benefit from the strategy described here. In our case, genetic testing performed before transfer did not permit definitive subtype classification.

Indications for aortic surgery in patients with Marfan syndrome are more stringent than in those without Marfan syndrome, with surgery often recommended when the aortic diameter reaches ≥ 45 mm, particularly in younger patients. Earlier intervention may be warranted at aortic diameters of approximately 40–45 mm in cases of rapid aortic enlargement (> 5 mm within 6 months) or with a family history of aortic dissection or rupture. Selection criteria for heart transplantation in patients with Marfan syndrome are similar to those for patients without Marfan syndrome. Candidates must have severe heart failure refractory to medical treatments, be < 65 years of age, and have no absolute or relative contraindications to transplantation. The point to emphasize is that even if residual dissection lesions do not reach the aforementioned borderline, replacing all lesions prior to heart transplantation is preferable for reducing the risk of future complications in patients with Marfan syndrome.

Our strategy for obtaining transplant eligibility for this patient was to address his comorbidities before being listed for a heart transplant, including an extended dissecting aortic aneurysm. We and the patient faced a significant hurdle: undergoing an aortic arch replacement and redo aortic valve replacement after previous aortic root replacement, followed by thoracoabdominal aortic replacement, while experiencing end-stage heart failure with an EF of 10%. This lengthy strategy to address all of his issues was apparently unachievable. Nonetheless, it was completed with the aid of centrifugal and pulsatile extracorporeal LVADs. Above all, thoracoabdominal aortic replacement with a centrifugal extracorporeal LVAD is by far the most invasive surgical procedure, associated with dreadful perioperative derangement of the coagulation system. We are not aware of any previous reports of patients undergoing TAAA repair with temporary extracorporeal LVAD support due to severely depressed cardiac function. The proof of the feasibility of this procedure is the originality of this case report.

In conclusion, we report a rare case of successful orthotopic heart transplantation performed after total aortic replacement in a patient with Marfan syndrome and end-stage heart disease. This strategy may reduce the risk of late vascular complications, a major prognostic factor in Marfan patients. Given that this is a single-institution case report, the generalizability of this strategy is limited, but complete resolution of extensive aortic disease before transplantation may help mitigate post-transplant vascular complications related to the underlying connective tissue disorder. 

## Supplementary Information

Supplementary Data 1. (a) Cardiac function pre- and post-transplantation (b) Cardiac catheterization findings pre- and post-transplantation (c) Renal function pre- and post-transplantation.

Supplementary Data 2. Timeline summarizing the entire clinical course.

## Data Availability

The datasets supporting the conclusions of this study are included in the article. Additional data and materials will be shared in accordance with the IRB protocol upon reasonable request.

## Abbreviations

ARDS: acute respiratory distress syndrome
AVR: aortic valve replacement
NPWT: negative-pressure wound therapy
CI: cardiac index
Cr: creatinine
EF: ejection fraction
eGFR: estimated glomerular filtration rate
LVAD: left ventricular assist device
LVDd / LVDs: left ventricular diastolic diameter / left ventricular systolic diameter
mPAWP: mean pulmonary arterial wedge pressure
mRAP: mean right atrial pressure
PAP: pulmonary artery pressure
SVI: stroke volume index
V-V ECMO: veno-venous extracorporeal membrane oxygenation
