# Supplementary material for: Successful orthotopic heart transplantation following total aortic replacement in a patient with Marfan syndrome: case report
Source: Gen Thorac Cardiovasc Surg Cases. 2026 Apr 17;5:22. doi: 10.1186/s44215-026-00249-2 (PMC13224456; doi:10.1186/s44215-026-00249-2)
Supplement: Supplementary file 1 — Supplementary Data 1. (a) Cardiac function pre- and post-transplantation (b) Cardiac catheterization findings pre- and post-transplantation (c) Renal function pre- and post-transplantation. [file 44215_2026_249_MOESM1_ESM.docx]

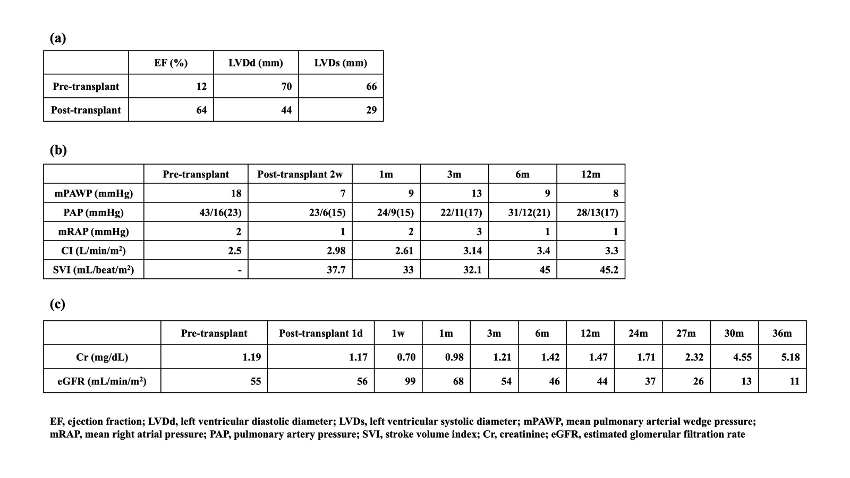


Supplementary data 1.

1. Cardiac function pre- and post-transplantation
2. Cardiac catheterization findings pre- and post-transplantation
3. Renal function pre- and post-transplantation
