# Supplementary figures and images for: Successful orthotopic heart transplantation following total aortic replacement in a patient with Marfan syndrome: case report
Source: Gen Thorac Cardiovasc Surg Cases. 2026 Apr 17;5:22. doi: 10.1186/s44215-026-00249-2 (PMC13224456; doi:10.1186/s44215-026-00249-2)

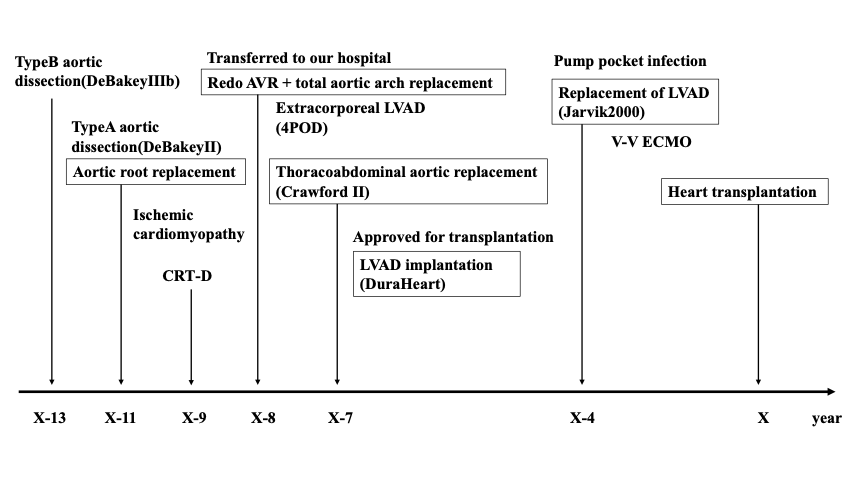


Supplementary data 2. Timeline summarizing the entire clinical course

Supplement: Supplementary file 2 — Supplementary Data 2. Timeline summarizing the entire clinical course. [file 44215_2026_249_MOESM2_ESM.docx]
